# Inference of financial networks using the normalised mutual information rate

**DOI:** 10.1371/journal.pone.0192160

**Published:** 2018-02-08

**Authors:** Yong Kheng Goh, Haslifah M. Hasim, Chris G. Antonopoulos

**Affiliations:** 1 Centre for Mathematical Sciences, Universiti Tunku Abdul Rahman, Kajang, Malaysia; 2 Department of Mathematical Sciences, University of Essex, Colchester, United Kingdom; Universidad Rey Juan Carlos, SPAIN

## Abstract

In this paper, we study data from financial markets, using the normalised Mutual Information Rate. We show how to use it to infer the underlying network structure of interrelations in the foreign currency exchange rates and stock indices of 15 currency areas. We first present the mathematical method and discuss its computational aspects, and apply it to artificial data from chaotic dynamics and to correlated normal-variates data. We then apply the method to infer the structure of the financial system from the time-series of currency exchange rates and stock indices. In particular, we study and reveal the interrelations among the various foreign currency exchange rates and stock indices in two separate networks, of which we also study their structural properties. Our results show that both inferred networks are small-world networks, sharing similar properties and having differences in terms of assortativity. Importantly, our work shows that global economies tend to connect with other economies world-wide, rather than creating small groups of local economies. Finally, the consistent interrelations depicted among the 15 currency areas are further supported by a discussion from the viewpoint of economics.

## Introduction

In finance, researchers use complex systems theory to understand the behaviour and dynamics of financial markets, as they can be regarded as complex systems with large numbers of interacting financial agents [1]. Treating financial markets as a complex system helps in understanding their relationship to other complex systems and using common approaches to study them. The interactions among the constituent parts in such systems are frequently non-linear.

Many approaches have been attempted to map the financial system as a network in which nodes stand for different financial agents and edges between the nodes for their interactions. Some of the methods used to investigate interactions across different financial agents include correlation-based networks, such as the minimal spanning tree approach introduced in [2]; the extension of the above approach proposed in [3]; the planar maximally filtered graph, which is also used in a recent study in [4]; and the average linkage minimum spanning tree proposed in [5]. Other approaches are based on the correlation threshold network [6] and partial correlation threshold network methods [7–9].

More recently, several econometric approaches have been applied to analyse the systematic spillover interconnectedness across multiple financial agents and infer the network structure. For example, the authors in [10] proposed a measure of return and volatility spillovers in the framework of vector autoregression (VAR) and generalised variance decomposition. Moreover, the authors in [11] employed the method in [10] to construct a volatility spillover network for measuring the connectedness of financial institutions. In the framework of Granger causality analysis, [12] proposes the use of Granger causality networks to quantify systemic risk in financial institutions in terms of mean spillovers, and recently the authors in [13] developed an extreme risk spillover network for analysing the interconnectedness across financial institutions.

It is thus important to study financial markets from the perspective of complex systems theory. The goal is to understand how these markets and their components are interrelated and how collective behaviours might emerge. Particularly, in our work, we consider time-series data from 15 world-wide financial markets, including the European Union (EU). Financial markets consist of components, such as currency exchange rates and stock indices. The data are the time-series of the 15 major currency exchange rates and stock indices of these financial markets. The components and their interrelations can be represented by a network of nodes and connections, where the nodes are either the currency exchange rates or stock indices, and the connections, or interactions among them, are usually non-linear. The relationships between the components and, consequently, between the markets, can be defined according to the amount of information exchanged between their respective time-series data. By doing so, we are able to quantify market interrelations and thus, analyse collective behaviours of different financial markets. The question whether these markets interact with themselves or influence each other is very important and useful to know to make informed decisions or choices. Here, we use a recently published information-mathematical method for network inference based on Mutual Information Rate (MIR) [14] to infer the structure of such networks. MIR is a measure of the amount of information exchanged per unit of time among stochastic sources or data sets [14, 15].

Mutual information (MI) and, particularly, MIR was first introduced by Shannon in 1948 as a “rate of actual transmission” of information [16] and was redefined more rigorously in [17] and later in [18]. It represents the MI exchanged per unit of time between two dynamical, correlated, variables and is based on mutual information which quantifies linear and non-linear interrelations between two systems or data sets. It is essentially a measure of how much information two systems, or two data sets, share. Even though MI is very important to understand various complex systems, ranging from the brain [19] to chaotic systems, there are three main difficulties that need to be overcome, namely: (a) MI in random memoryless processes does not consider the degree of memory that financial markets have been proved to contain [20–22]; (b) it is necessary to determine what is considered a significant event in the complex system under study as the probabilities of significant events often need to be known prior to the calculation of MI; and (c) due to the usually limited size of data sets, which introduces finite-size effects, it is complicated to calculate these probabilities accurately. This might lead to a biased calculation of MI [23]. Other limitations might come by the use of linear measures (e.g. correlation measures), as they ignore the complexity present in such systems and the fact that financial markets present non-linear behaviours with regard to stock returns. In [24, 25], the authors overcame the above limitations by using partial mutual information. Another way to overcome such challenges has been proposed in [15, 20], where the authors calculate the amount of information exchanged per unit of time between two nodes in a dynamical network, i.e. MIR, as it permits a more reliable measure of the hierarchical dependency in networks.

Previous studies focused on using MIR in single financial components, such as stocks traded on the New York Stock Exchange (NYSE) [20, 24] and Shanghai Stock Exchange markets [25]. In our study, we are interested in identifying interrelations among nodes in the financial networks of the 15 currency exchange rates and stock indices [20], and not in inferring the directionality of information exchange among them, i.e. causality. We thus assume throughout the paper that all financial networks are undirected, i.e. connections between pairs of nodes are bidirectional, and that such connections are due to their interactions. We focus on demonstrating how one can use the normalised MIR [14] to infer the network structure in financial-markets data and, particularly, the connectivity among the nodes of the network of the 15 currency exchange rates and stock indices. Our results show that the inferred networks of the currency exchange rates and stock indices are small-world networks, sharing similar properties and having differences in terms of assortativity [26]. Importantly, our work shows that global economies tend to connect with other economies world-wide, rather than creating small groups of local economies. Finally, the consistent interrelations depicted among the 15 currency areas are further supported by a discussion from the economics viewpoint.

## Materials and methods

### Information and network connectivity

As is well-known, a system can produce information which can be transferred among its parts [15, 19, 27–31]. When information is transferred, there are at least two components involved that are physically interacting in either direct or indirect, and linear or non-linear ways. These components can be either time-series data, modes, or related functions, and from the mathematical viewpoint, they are defined on subspaces or projections of the state space of the system [15, 31].

In this work, we will use a quantity based on MIR to study the amount of information transferred per unit of time between any two components of a system, namely to determine whether a connection exists between these components. Such an existence means there is a bidirectional connection between them attributed to their interaction. Particularly, MIR measures the amount of information exchanged per unit of time between two non-random, correlated variables; its application to time-series data is of primordial importance and will be used to determine if a bidirectional connection exists between any two nodes in the system. In this framework, the strength of such connections can also be inferred, in the sense that they will be compared to those from all other pairs of nodes in the network, so long as the available data collected from the financial markets are sufficient in numbers to allow for discrimination between stronger and weaker connections.

### Mutual information

MI and MIR were originally introduced by Shannon in 1948 [16]. In particular, the MI of two random variables, *X* and *Y*, is a measure of their mutual dependence and quantifies the “amount of information” obtained about one random variable *X*(*Y*), after observing another random variable *Y*(*X*). It is defined by [16, 32]

$$\begin{array}{c} I_{XY}\left(N\right)=H_{X}+H_{Y}-H_{XY}, \end{array}$$ (1)

where *N* is the total number of random events in *X* and *Y*. *H*_*X*_, and similarly *H*_*Y*_, are the marginal entropies of *X* and *Y* (i.e. the Shannon entropies) respectively, defined by

$$\begin{array}{c} H_{X}=-\sum_{i=1}^{N}P_{X}\left(i\right)\log P_{X}\left(i\right) \end{array}$$ (2)

where *P*_*X*_(*i*) is the probability of a random event *i* happening in *X*.

The joint entropy, *H*_*XY*_ in Eq (1) measures how much uncertainty there is in *X* and *Y* when taken together. It is defined as

$$\begin{array}{c} H_{XY}=-\sum_{i=1}^{N}\sum_{j=1}^{N}P_{XY}\left(i,j\right)\log P_{XY}\left(i,j\right), \end{array}$$ (3)

where *P*_*XY*_(*i*, *j*) is the joint probability of both events *i* and *j* occurring simultaneously in variables *X* and *Y*.

Equivalently, we can define MI by

$$\begin{array}{c} I_{XY}\left(N\right)=\sum_{i=1}^{N}\sum_{j=1}^{N}P_{XY}\left(i,j\right)\log\left(\frac{P_{XY}\left(i,j\right)}{P_{X}\left(i\right)P_{Y}\left(i\right)}\right). \end{array}$$ (4)

This equation provides a measure of the strength of the dependence between the two random variables, and the amount of information *X* contains about *Y*, and vice-versa [32]. When MI is zero, *I*_*XY*_ = 0, the strength of the dependence is null and thus *X* and *Y* are independent: knowing *X* does not give any information about *Y* and vice-versa. Note also that MI is symmetric, i.e. *I*_*XY*_ = *I*_*YX*_ and thus cannot be used to study causality between *X* and *Y*.

The computation of *I*_*XY*_(*N*) from time-series data requires the calculation of probabilities in a suitably defined probability space on which a partition based on the *N*^2^ events can be defined (see for example Eqs (3) and (4)). Particularly, the probability space is a 2-dimensional space defined by the values of *X* (horizontal axis) and *Y* (vertical axis). The probabilities in this space are defined in terms of the frequency of occurrence of the events over all events in the 2-dimensional space and thus, what will be considered as an event is crucial for the definition of the probability space and its partition. *I*_*XY*_ can be computed for any pair of nodes, *X* and *Y*, in the same network and can then be compared with *I*_*XY*_ of any other pair of nodes in the same network. However, *I*_*XY*_ is not suitable for comparisons when it comes from different systems as it is possible for different systems to have different correlation decay-times and time-scales [33–35] in their dynamical evolution.

There are various methods to compute MI, depending on the method used to calculate the probabilities in Eq (4). The main methods are the bin method [36], the density-kernel method [37], and the estimation of probabilities from distances between closest neighbours [38]. In this work, we use the bin method, and particularly, grids of *N*^2^ equally-sized cells (grids of size *N*) [14]. This method tends to overestimate MI because of the finite length of recorded time-series data, and the finite resolution of non-Markovian partitions [23, 39]. However, these errors are systematic and always present for any given non-Markovian partition as in our work. To avoid such errors, we apply the two normalisations proposed in [14] to calculate the probabilities in Eq (4).

### Mutual Information Rate

The Mutual Information Rate (MIR) came about as a method to bypass problems associated with the resolution of non-Markovian partitions, specifically in calculating MI for such partitions. In [15], it was shown how to calculate MIR for two finite length time-series, irrespective of the partitions in the probability space. MIR is invariant with respect to the resolution of Markov partitions and is defined by

$$\begin{array}{ccc} {\text{MIR}}_{XY} & = & \lim_{N\to \infty }\lim_{L\to \infty }\sum_{i=1}^{L-1}\frac{I_{XY}\left(i+1,N\right)-I_{XY}\left(i,N\right)}{L}\\ & = & \lim_{N\to \infty }\lim_{L\to \infty }\frac{I_{XY}\left(L,N\right)-I_{XY}\left(1,N\right)}{L}\\ & = & \lim_{N\to \infty }\lim_{L\to \infty }\frac{I_{XY}\left(L,N\right)}{L}, \end{array}$$ (5)

where *I*_*XY*_(*L*, *N*) represents the MI of Eq (1) between two random variables *X* and *Y*, considering trajectories of length *L* that follow an itinerary over cells in a grid of infinitely many cells *N*. Note that *I*_*XY*_(1, *N*)/*L* tends to zero in the limit of infinitely long trajectories, i.e. when *L* → ∞.

For finite-length time-series *X* and *Y*, the definition in Eq (5) can be further reduced, as demonstrated in [15], to

$$\begin{array}{c} {\text{MIR}}_{XY}=\frac{I_{XY}\left(N\right)}{T(N)}, \end{array}$$ (6)

where *I*_*XY*_(*N*) is defined as in Eqs (1) and (4), and *N* is the number of cells in a Markov partition of order *T* for a particular grid-size *N*.

It is important to note that, while *T* and *N* are both finite, for statistically significant results, a sufficiently large number of data in the time-series is required to ensure that the length of the time-series is sufficiently larger than *T*, and thus a more saturated distribution of data can be achieved across the probability space and its partitions.

## Demonstration of the method

We first tested the method by attempting to reproduce the structure of known networks where the dynamics in their nodes is given by chaotic maps, before applying it to data from financial markets. Particularly, we start with networks with given binary adjacency matrices (which we call original adjacency matrices) to allow for comparisons between the original and the inferred one. By binary, we mean two nodes in the network can either be directly connected, which corresponds to an entry equal to 1 in the matrix, or unconnected, which corresponds to a 0 entry. Moreover, since the connections are considered bidirectional, the adjacency matrices will be symmetric. We then couple the chaotic maps according to the original adjacency matrices and record the evolution of their dynamics and produce time-series data for each node in the original network. Next, we input these time-series data to the proposed method, which produces an inferred, binary adjacency matrix. To quantify the percentage of successful inference, we subtract the original from the inferred adjacency matrix. If the resulting matrix is the zero matrix, then we call this 100% successful network inference. Should there be spurious or missed connections, this difference would not be the zero matrix, and thus would correspond to a smaller percentage. For example, 100% successful inference means that the proposed method infers correctly all connections in the original network used to produce the recorded data, with no spurious or missed connections.

### Circle map network

**Fig 1:**
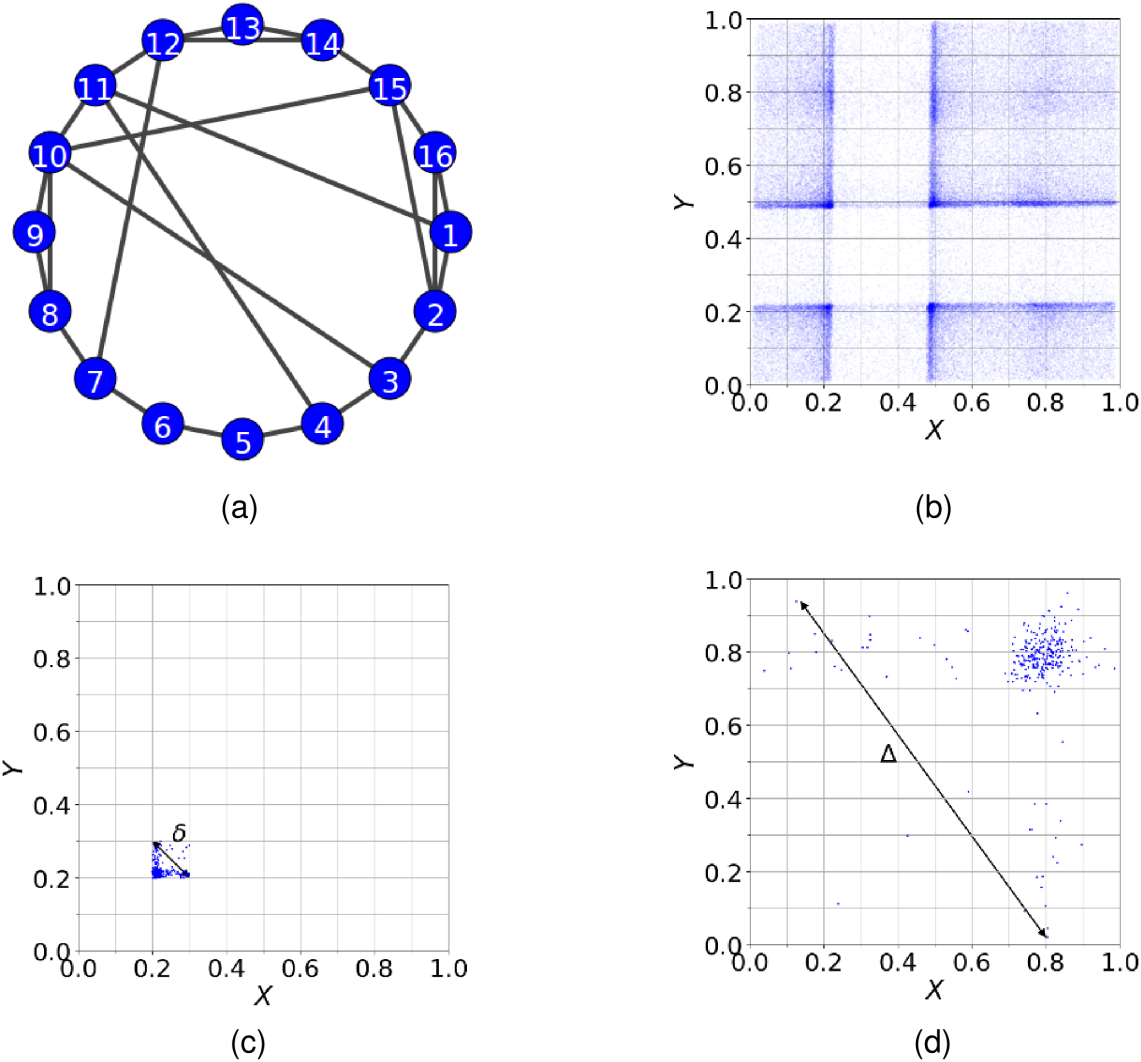
The CMN, distribution of data points and expansion of points in Ω. Panel (a): The CMN is composed of 16 coupled nodes as shown by its network. The dynamics in each node is given by Eqs (7) and (8). Panel (b): The distribution of points in Ω obtained from Eqs (7) and (8), plotted in a 10 × 10 grid of equally-sized cells. Panel (c): The points belong initially to a cell of the same grid and expand to a larger extend of Ω after three iterations of the dynamics, occupying more than one cells. *δ* is the maximum distance in the initial cell and Δ the maximum distance after the points have expanded to a larger extend of Ω.

Following [14], we first apply the method to the circle map network (CMN). The network itself is shown in Fig 1(a) and is composed of 16 coupled nodes with dynamics given by [40]

$$\begin{array}{c} x_{n+1}^{i}=\left(1-\alpha \right)f\left(x_{n}^{i},r\right)+\frac{\alpha }{k_{i}}\sum_{j=1}^{M}A_{ij}f\left(x_{n}^{j},r\right), \end{array}$$ (7)

where *M* = 16 is the total number of nodes, $x_{n}^{i}$ the *n*-th iterate of map *i* = 1, 2, …, *M* and *α* ∈ [0, 1] the coupling strength. (*A*_*ij*_) is the binary adjacency matrix of the network in Fig 1(a). $k_{i}=\sum_{j=1}^{M}A_{ij}$ is the node-degree, *r* the parameter of each map, and *f*(*x*_*n*_, *r*) the circle map, defined by

$$\begin{array}{c} f\left(x_{n},r\right)=x_{n}+r-\frac{K}{2\pi }\sin\left(2\pi x_{n}\right)\;\mathrm{mod}\;1. \end{array}$$ (8)

For the parameters in Eq (7), we follow [14] and use *α* = 0.03 to create a weakly interacting system and, *r* = 0.35 and *K* = 6.9115 that correspond to fully developed chaos for each individual map. $x_{0}^{i}$ is initialised randomly, and the transient period of the first 1,000 iterations is discarded keeping the next 100,000 iterations generated by the dynamics in the network, which we recorded to produce the time-series data.

We then calculate MI and MIR for a pair of nodes *X*, *Y* by defining a 2-dimensional probability space Ω formed by the time-series *X* and *Y* (see Fig 1(b)). Ω is partitioned into a grid of *N* × *N* equally-sized cells (following the bin method [14, 36]) where the probability of an event *i* in *X* is

$$\begin{array}{c} P_{X}\left(i\right)=\frac{\text{number}\;\text{of}\;\text{data}\;\text{points}\;\text{in}\;\text{column}\;i}{\text{total}\;\text{number}\;\text{of}\;\text{data}\;\text{points}\;\text{in}\;\Omega }, \end{array}$$ (9)

and that of an event *j* in *Y* is

$$\begin{array}{c} P_{Y}\left(j\right)=\frac{\text{number}\;\text{of}\;\text{data}\;\text{points}\;\text{in}\;\text{row}\;j}{\text{total}\;\text{number}\;\text{of}\;\text{data}\;\text{points}\;\text{in}\;\Omega }. \end{array}$$ (10)

Similarly, the joint probability can be defined by the ratio of points in cell (*i*, *j*) of the same partition in Ω and is expressed by

$$\begin{array}{c} P_{XY}\left(i,j\right)=\frac{\text{number}\;\text{of}\;\text{data}\;\text{points}\;\text{in}\;\text{cell}\;(i,j)}{\text{total}\;\text{number}\;\text{of}\;\text{data}\;\text{points}\;\text{in}\;\Omega }. \end{array}$$ (11)

MI therefore can be calculated from Eq (4) for different values of grid sizes *N*, and is thus partition-dependent as it gives different values for different grid sizes.

To ensure there is always a sufficiently large number of data points in the cells of the *N* × *N* partition of Ω, we require that the average number of points in all occupied cells be sufficiently larger than the number of occupied cells,

$$\begin{array}{c} \left\langle N_{0}\left(N\right)\right\rangle \geq N_{oc}, \end{array}$$ (12)

where *N*_*oc*_ is the number of occupied cells and 〈*N*_0_(*N*)〉 is the average number of points in all occupied cells in Ω. For the CMN, we have used a data set of 100,000 points which guarantee that this condition is satisfied for grid sizes up to *N*_max_ = 19. Based on this consideration, we have calculated *I*_*X*,*Y*_ for grid sizes ranging from 0.2*N*_max_ to *N*_max_.

In order to compute MIR using Eq (6), we need to estimate the correlation decay time *T*(*N*), which is a special time that represents the time for the dynamical system (or data sets *X* and *Y*) to lose memory from the initial state or the correlation to decay to zero. In systems with sensitivity to initial conditions, i.e. chaotic systems, predictions are only possible for times smaller than *T*(*N*) and thus, after this time the system becomes unpredictable. It can be calculated in different ways, e.g. by using the diameter of an associated itinerary graph *G* [14], or the Lyapunov exponents of the dynamics or the largest expansion rates [15]. All these ways exploit the fact that the dynamics is chaotic and thus, the property that the points expand to the whole extend of Ω after about *T*(*N*) time for a particular grid-size *N*.

In this work, we estimate *T*(*N*) by the largest expansion rate *e*_1_, which is easy to compute from data sets. *T*(*N*) is difficult to calculate in practical situations or even in toy-model dynamical systems, as the Markov partitions are unknown and difficult to define. Thus, we exploit the fact that a necessary condition to determine the shortest time for the correlation to decay to zero is the time it takes to points in cells of Ω to expand and cover completely Ω. Particularly,

$$\begin{array}{c} T\left(N\right)\approx \frac{1}{\lambda_{1}}\log\frac{1}{N}, \end{array}$$ (13)

where *λ*_1_ is the largest positive Lyapunov exponent of the dynamics in the network [41]. However, in a system or for recorded data sets for which *λ*_1_ cannot be estimated or computed, it can be replaced by the largest expansion rate *e*_1_, defined by

$$\begin{array}{c} e_{1}=\frac{1}{N_{oc}}\sum_{i=1}^{N_{oc}}\frac{1}{t}\log L_{1}^{i}\left(t\right), \end{array}$$ (14)

with *e*_1_ ≤ *λ*_1_ in general. The equality holds when the system has constant Jacobian, is uniformly hyperbolic, and has a constant natural measure [15]. However, when dealing with real data for which the equations of motion are unknown, it is hardly possible to know or prove these assumptions mathematically. Thus, one approximates the maximum Lyapunov exponent *λ*_1_ by computing *e*_1_, which is easier to estimate, whereas Lyapunov exponents demand more computational effort. As shown in [14, 15] and here in, this approximation works well in terms of successful network inference in dynamical systems governed by equations of motion, and for real data for which the equations of motion are unknown.

In Eq (14), $L_{1}^{i}\left(t\right)$ is the largest distance between pairs of points in cell *i* at time *t* divided by the largest distance between pairs of points in cell *i* at time 0, and is expressed as

$$\begin{array}{ccc} L_{1}^{i}\left(t\right) & = & \frac{\Delta }{\delta }\\ & = & \frac{\text{largest}\;\text{distance}\;\text{between}\;\text{pairs}\;\text{of}\;\text{point}\;\text{in}\;\text{cell}\;i\;\text{at}\;\text{time}\;t}{\text{largest}\;\text{distance}\;\text{between}\;\text{pairs}\;\text{of}\;\text{point}\;\text{in}\;\text{cell}\;i\;\text{at}\;\text{time}\;0}. \end{array}$$ (15)

In Fig 1(c) and 1(d), we present an example of the expansion of points for the CMN that initially belong to a single cell (Fig 1(c)) and after three iterations of the dynamics, expand to a larger portion of Ω (Fig 1(d)). In Fig 1(c), we denote by *δ* the maximum distance for a pair of points in the initial cell, and in Fig 1(d) by Δ the maximum distance after they have expanded to a larger extend of Ω.

In the calculation of MIR, since we are using partitions of fixed-size cells which are non-Markovian, errors will occur, causing a systematically biased computation towards larger MIR values, making MIR partition-dependent. To account for this, and to make MIR partition-independent, the authors in [14] came up with the following two normalisations.

Particularly, there is a systematic error coming from the non-Markovian nature of the equally-sized cells in the grids under consideration, as a smaller *N* is more likely to create a partition which is significantly different from a Markovian one than a larger grid size *N*. Moreover, using the fact that MIR_*XY*_ = MIR_*YX*_ and MIR_*XX*_ = 0, we can narrow the number of *X*, *Y* pairs from *M*^2^ to *M*(*M* − 1)/2. The method presented in [14] to avoid such errors is to normalise MIR for each grid size *N* as follows: For fixed *N*, MIR_*XY*_(*N*) is first computed for all *M*(*M* − 1)/2 pairs of nodes and is normalised with respect to their minimum and maximum values. The reason is that for unconnected pairs of nodes, MIR is numerically very close to zero, and doing so the new ${\hat{\text{MIR}}}_{XY}$ in Eq (16) will be in the interval [0, 1]. This normalisation can be achieved by computing

$$\begin{array}{c} {\hat{\text{MIR}}}_{XY}\left(N\right)=\frac{{\text{MIR}}_{XY}\left(N\right)-\min\left\{{\text{MIR}}_{XY}\left(N\right)\right\}}{\max\left\{{\text{MIR}}_{XY}\left(N\right)\right\}-\min\left\{{\text{MIR}}_{XY}\left(N\right)\right\}}, \end{array}$$ (16)

where MIR_*XY*_(*N*) is the MIR of nodes *X* and *Y*, min{MIR_*XY*_(*N*)} is the minimum MIR of all *M*(*M* − 1)/2 pairs, and similarly max{MIR_*XY*_(*N*)} is the maximum MIR over all MIR values of the *M*(*M* − 1)/2 pairs.

Moreover, since we use ${\hat{\text{MIR}}}_{XY}\left(N\right)$ for a range of grid sizes *N*, we can further normalise ${\hat{\text{MIR}}}_{XY}$ by [14]

$$\begin{array}{c} {\bar{\text{MIR}}}_{XY}=\frac{\sum_{i}{\hat{\text{MIR}}}_{XY}\left(N_{i}\right)}{\max\{\sum_{i}{\hat{\text{MIR}}}_{XY}\left(N_{i}\right)\}}, \end{array}$$ (17)

where the maximum is now taken over the *N*_*i*_ grid sizes considered in [0.2*N*_max_, *N*_max_]. This normalisation ensures again that ${\bar{\text{MIR}}}_{XY}$ values are in [0, 1].

To infer a network using Eq (17), we fix a threshold *τ* ∈ [0, 1] and consider the pair *XY* as connected if ${\bar{\text{MIR}}}_{XY}\geq \tau$. If so, then the corresponding entry in the adjacency matrix *A*^*c*^ of the inferred network becomes 1, i.e. $A_{XY}^{c}=1$ or 0 if ${\bar{\text{MIR}}}_{XY}<\tau$ as the pair is considered as unconnected.

**Fig 2:**
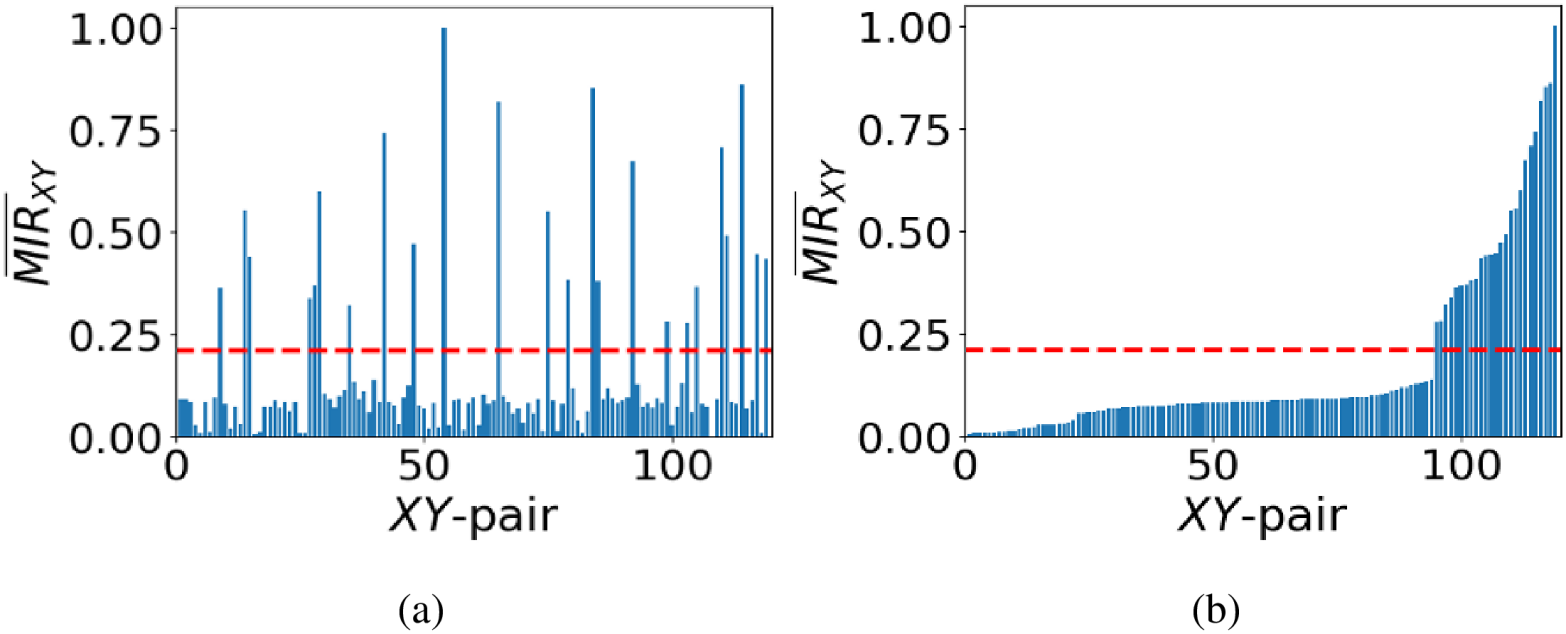
Estimation of the threshold τ for the inference of CMN. Panel (a) is the plot of ${\bar{\text{MIR}}}_{XY}$ before ordering its values in ascending order, and (b) after ordering them in ascending order. Following the approach in the text, *τ* ≈ 0.21 plotted by the red dash line in both panels.

The choice of an appropriate *τ* is therefore crucial in depicting successfully the structure of the original network from the recorded data. If *τ* is set too high, real connections among nodes might be missed, while if set too low, spurious connections between nodes might appear in the inferred network. The problems with setting *τ* stem from the above normalisations. To address this, we follow [42] and determine *τ* by first sorting all ${\bar{\text{MIR}}}_{XY}$ values in ascending order, and then identifying the first *XY* pair for which ${\bar{\text{MIR}}}_{XY}$ increases more than 0.1 —which accounts for an abrupt change in ${\bar{\text{MIR}}}_{XY}$ values —and then set the threshold *τ* as the middle value of the ${\bar{\text{MIR}}}_{XY}$ for the identified pair and that for the immediately previous pair. This is based on the observation that there are two main groups of ${\bar{\text{MIR}}}_{XY}$ values, i.e. one for the connected and one for the unconnected nodes in the network (see Fig 2(b)).

We demonstrate the application of this approach for network inference in Fig 2. Particularly, in panel (a), we plot the ${\bar{\text{MIR}}}_{XY}$ values for all pairs *XY* before ordering them in ascending order, and in (b) after ordering them in ascending order. It is evident in panel (b) that there are two groups of ${\bar{\text{MIR}}}_{XY}$ values, namely one for the unconnected pairs (left side of the plot with relatively small values) and another one for the group of connected pairs (right side of the plot with relatively large values), separated by an abrupt change in ${\bar{\text{MIR}}}_{XY}$ values. Following the method for setting *τ*, we have estimated that *τ* ≈ 0.21 (plotted by the red dash line in both panels), which crosses the bar for which there is an abrupt change. This leads to the 100% successful inference of the original network as shown in Fig 1(a).

## Inferring networks using data from additional sources

### Introducing an additional node with uniform, uncorrelated, random data

One of the main difficulties in inferring the structure of a network is the estimation of *τ*. The notion of an abrupt change might be subjective (it might be considered one change or even more than one abrupt changes), especially when dealing with real data or when the number of nodes in the network is large. Here, we introduce another approach to improve network inference by setting *τ* according to the ${\bar{\text{MIR}}}_{XY}$ value of a pair of nodes with data with known properties, which are disconnected from the network. This ${\bar{\text{MIR}}}_{XY}$ value is then used to set the threshold for network inference.

To demonstrate this, we will use uniformly random, uncorrelated, data as an additional source. The rationale is that one would expect that the ${\bar{\text{MIR}}}_{XY}$ and correlation decay time *T*, between any two nodes of a network of such data, would be close to zero and one, respectively. This idea can be appreciated by using a network of isolated nodes. To this end, we set *α* = 0 in Eq (7), use 6 nodes (*N* = 6) and choose the logistic map in its chaotic regime (i.e. *f*(*x*_*n*_, *r* = 4)),

$$\begin{array}{c} f_{i}\left(x_{n}^{I}\right)=4x_{n}^{i}\left(1-x_{n}^{i}\right). \end{array}$$ (18)

**Fig 3:**
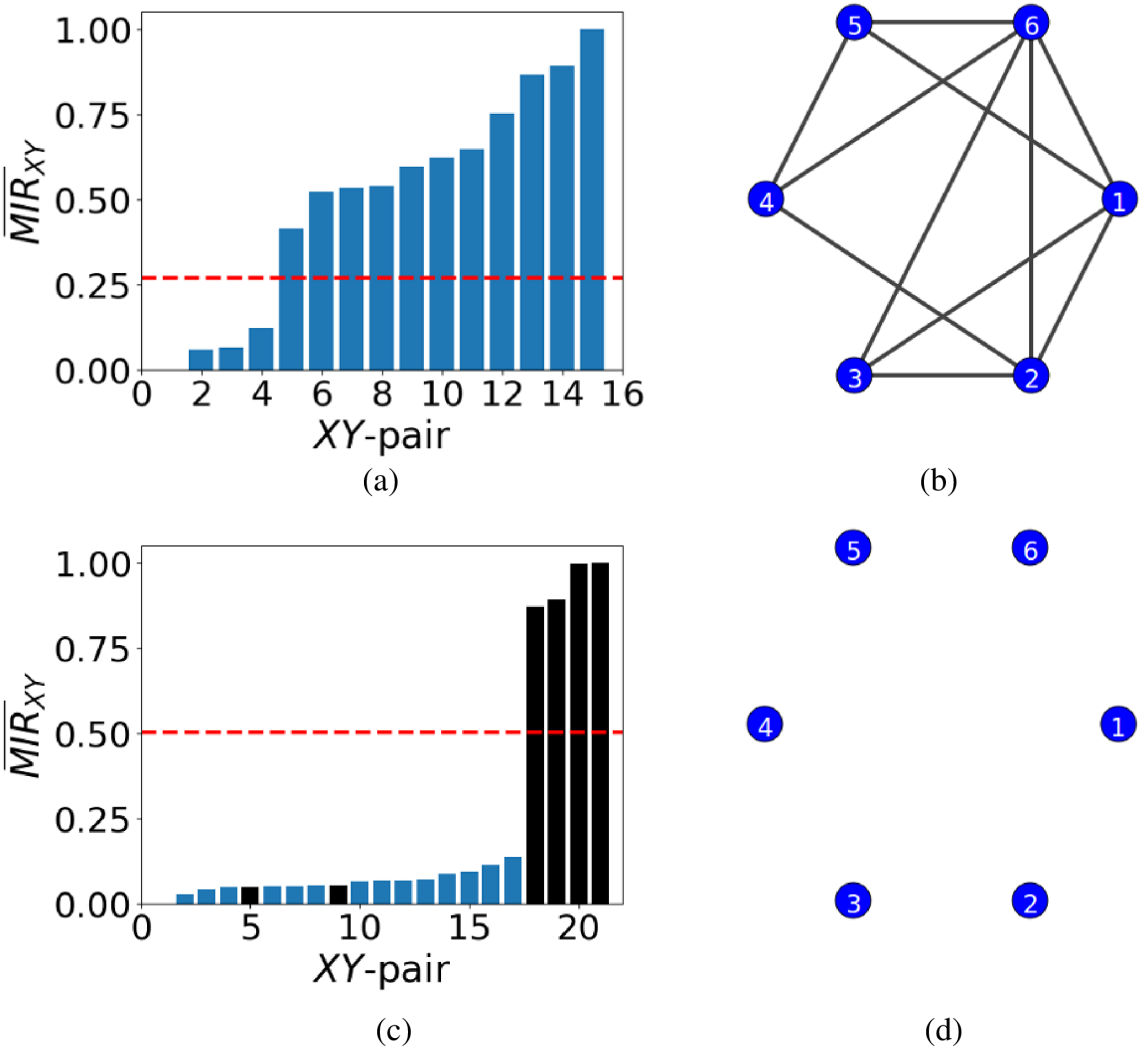
Results for the case of additional data for a pair of nodes with uniformly random, uncorrelated data. Panel (a) shows the estimation of *τ* ≈ 0.27 based on the ordered ${\bar{\text{MIR}}}_{XY}$ values for a network of 6 isolated nodes without the introduction of the pair of nodes with uniformly random, uncorrelated data. Panel (b) is the unsuccessfully inferred network based on panel (a). Panel (c) shows the ordered ${\bar{\text{MIR}}}_{XY}$ values for the same network with the nodes of random data added. The black bars are the ${\bar{\text{MIR}}}_{XY}$ values that come from the pair of additional nodes. Panel (d) shows the resulting successfully inferred network of isolated, disconnected nodes. In panels (a) and (c), we plot *τ* by a red dash line where *τ* ≈ 0.27 in (a) and *τ* ≈ 0.5 in (c).

The normalisation process casts the ${\bar{\text{MIR}}}_{XY}$ values in [0, 1] for all pairs of nodes *XY*. Often, the resulting ordered ${\bar{\text{MIR}}}_{XY}$ values do not allow for a clear determination of *τ* as evidenced in Fig 3(a). Without the use of an additional source of uniformly random, uncorrelated data for a pair of nodes disconnected from the rest of the network, one would compute *τ* ≈ 0.27 and would obtain the result in the inferred network in panel (b), which is clearly not the original one (seen in panel (d)) as the network in panel (b) consists of only isolated, disconnected nodes! In contrast, when using uniformly random, uncorrelated data for a pair of nodes disconnected from the rest of the network, and applying the same method, panel (c) shows an abrupt change in the ordered ${\bar{\text{MIR}}}_{XY}$ values that can be exploited to infer the network structure. In this case, *τ* ≈ 0.5 which leads to the successful network inference shown in panel (d). The black bars correspond to the ${\bar{\text{MIR}}}_{XY}$ values for the pair of introduced nodes with other nodes in the network.

### Indirect information exchange and bidirectional connections

**Fig 4:**
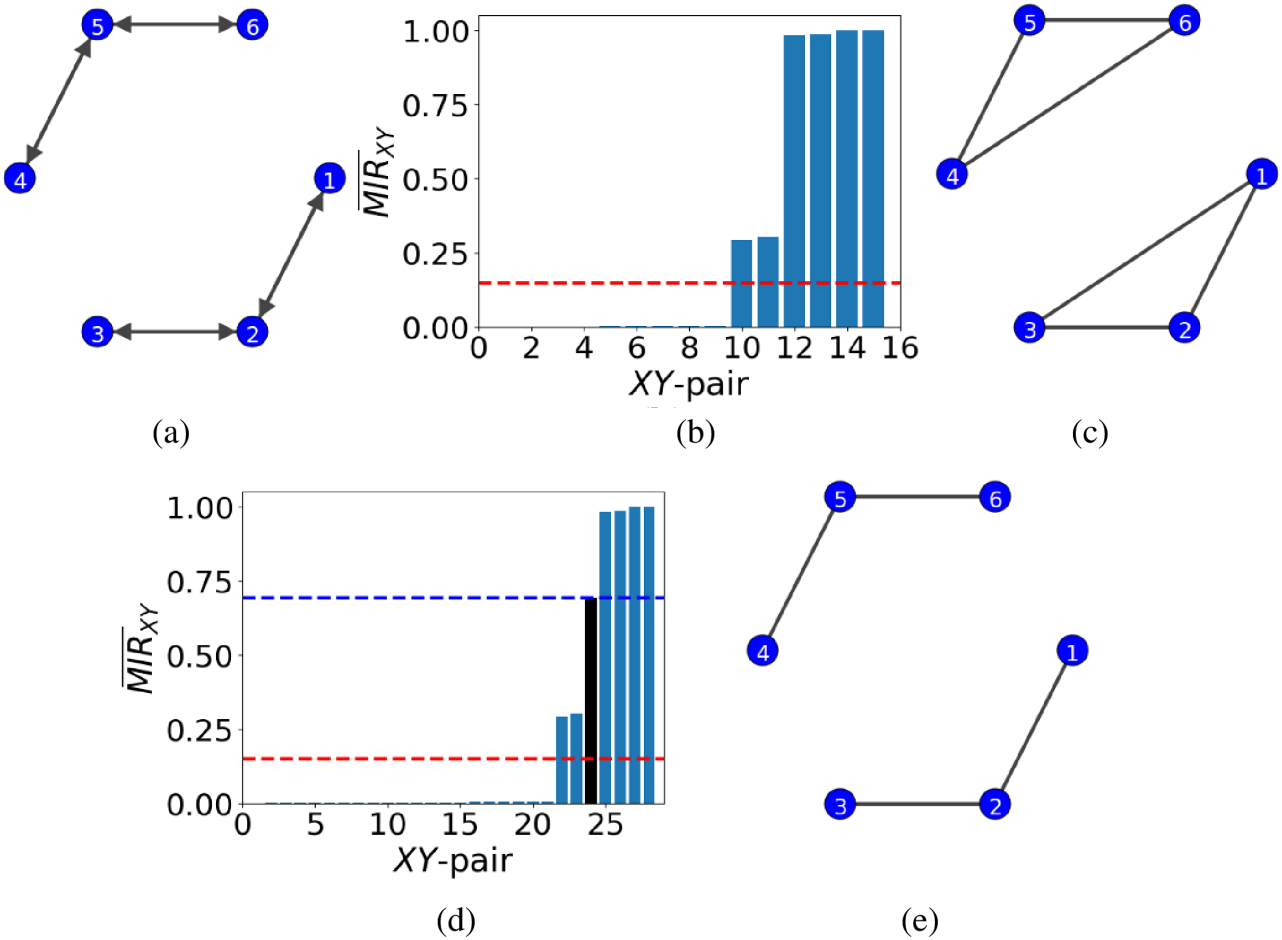
Network inference in the case of two, disconnected, triplets of nodes. Panel (a) shows the network of weakly coupled (*α* = 0.1) logistic maps. Nodes 1 and 3 interact indirectly through node 2, and similarly, nodes 4 and 6 through node 5. Panel (b) shows the ordered ${\bar{\text{MIR}}}_{XY}$ and threshold *τ* (red dash line) when no additional nodes are introduced to the network. The red dash line corresponds to *τ* ≈ 0.16. Panel (c) shows the unsuccessfully inferred network when using only the data from the network in panel (a). Panel (d) shows the ordered ${\bar{\text{MIR}}}_{XY}$ values for the same network as in (a) with the additional data from the pair of directed nodes (see text). The blue dash line represents the threshold computed for the pair of directed nodes (*τ* ≈ 0.69) and the red dash line corresponds to *τ* ≈ 0.16 from panel (b). Finally, panel (e) shows the successfully inferred network by considering as connected nodes only those with ${\bar{\text{MIR}}}_{XY}$ bigger than the blue dash threshold.

Next, we will examine a network of six weakly coupled (*α* = 0.1) logistic maps (18) seen in Fig 4(a) that corresponds to the binary adjacency matrix

$$\begin{array}{c} \left(A_{ij}\right)=\left[\begin{array}{cccccc} 0 & 1 & 0 & 0 & 0 & 0\\ 1 & 0 & 1 & 0 & 0 & 0\\ 0 & 1 & 0 & 0 & 0 & 0\\ 0 & 0 & 0 & 0 & 1 & 0\\ 0 & 0 & 0 & 1 & 0 & 1\\ 0 & 0 & 0 & 0 & 1 & 0 \end{array}\right]. \end{array}$$

This network consists of two triplets of nodes which are disconnected from each other. In each triplet, the two end nodes do not exchange information directly, but only indirectly through the intermediate nodes (i.e. 2 and 5, respectively). Applying the proposed method for network inference without the use of any additional nodes will result in inferring the indirect exchange of information as a direct one, where *τ* ≈ 0.16, depicted as the red dash line in Fig 4(b), leading to a spurious direct connection between the end nodes (see Fig 4(c)).

The addition of a new pair of nodes with uniformly random, uncorrelated data disconnected from the rest of the network will not help either in inferring successfully the network structure as the data from the chaotic dynamics are also uncorrelated in time. An alternative approach is to add a pair of directed nodes to the network, again disconnected from the main network. These nodes will be represented by chaotic logistic maps (i.e. with *r* = 4 and *α* = 0.1 in Eq (18)) with the adjacency matrix

$$\begin{array}{c} \left(A_{ij}\right)=\left[\begin{array}{cc} 0 & 1\\ 0 & 0 \end{array}\right]. \end{array}$$

Only the second node is coupled to the first and thus the information exchange is unidirectional from the second to the first, and the ${\bar{\text{MIR}}}_{XY}$ would not be as high as for a bidirectional connection. Consequently, we may assume that information exchange smaller than ${\bar{\text{MIR}}}_{XY}$ for this particular pair will not be considered as a connection and will be represented by 0 in the inferred adjacency matrix (see Fig 4(d) where the new threshold, depicted as the blue dash line, is now set at *τ* ≈ 0.69 and the old one is represented by the red dash line at *τ* ≈ 0.16). Following the proposed method for network inference for the augmented data and for *τ* ≈ 0.69, we arrive at the 100% successfully inferred network in Fig 4(e) which is the same as in Fig 4(a).

### Application to correlated normal-variates data

Since the global financial-markets data of different currency areas are correlated [43], an interesting application of the proposed method would be to correlated normal-variates data. In most cases, real data have no obvious dynamical system equations to help relate the different variables involved. For example, global financial markets of different countries are correlated, but the underlying equations that govern their evolution are unknown [43].

**Fig 5:**
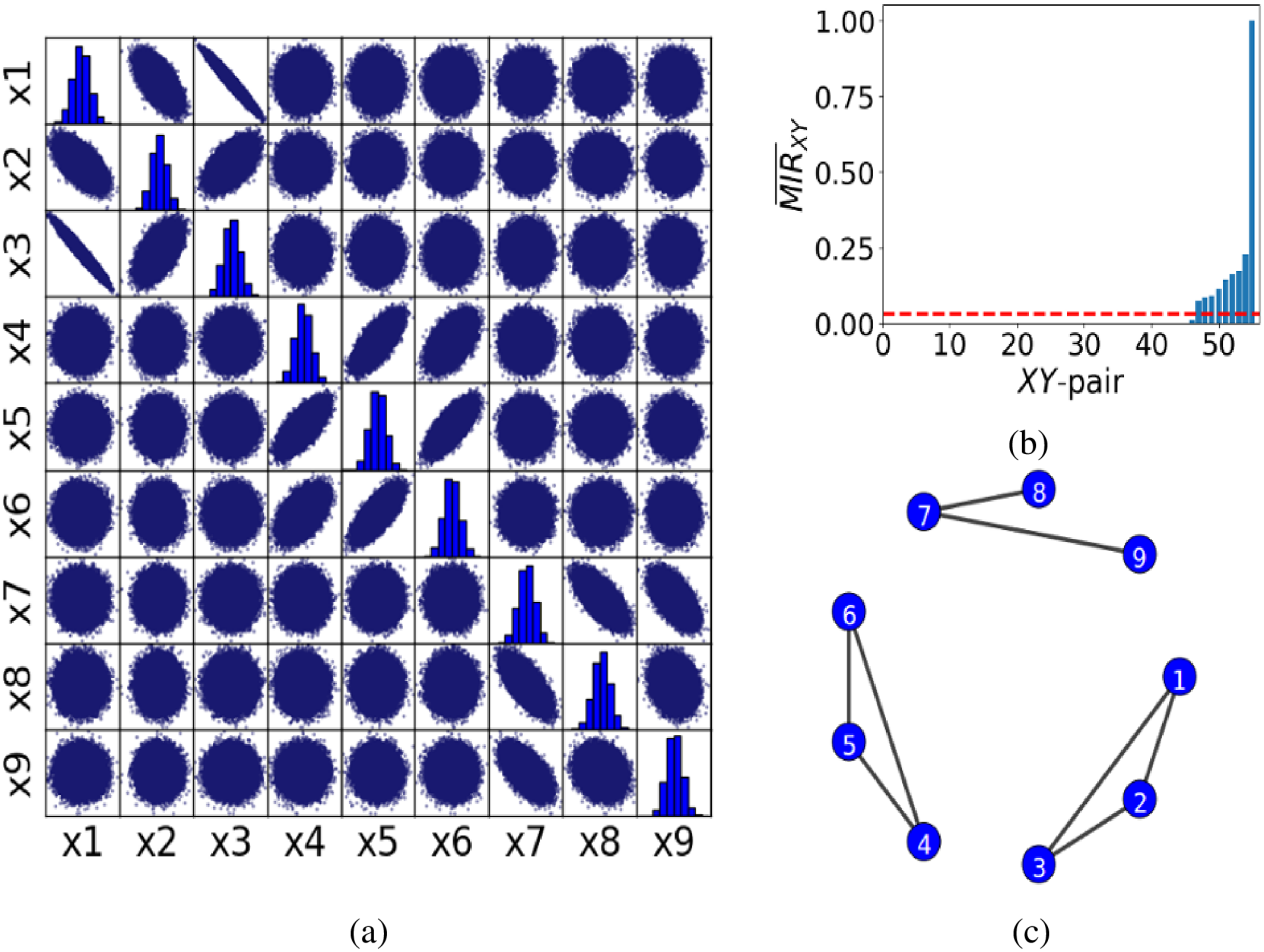
Application of the proposed method to correlated normal-variates data. Panel (a) shows the scatter matrix of nine data sets split into three groups (first group: x1, x2, x3, second group: x4, x5, x6 and third group: x7, x8, x9). Each group consists of three correlated normal-variates with zero correlation among the groups. Fig 5(a) shows the scatter matrix of the three groups of data (x1, …, x9). The circular pattern indicates that the two nodes are independent (or weakly correlated), whereas an elongated one shows strong correlation, either positive or negative depending on the orientation of the pattern. Panel (b) is the ordered ${\bar{\text{MIR}}}_{XY}$ for the correlated variates. The red dash line corresponds to *τ* ≈ 0.06. Panel (c) shows the successfully inferred network resulting from (b).

To demonstrate this, we generated three groups of correlated normal-variates data. Each group *i* = 1, 2, 3 consists of three correlated normal-variates data specified by a covariance matrix Σ_*i*_ with the three groups being uncorrelated with each other. Fig 5(a) shows the scatter matrix of the three groups of data (first group: x1, x2, x3, second group: x4, x5, x6 and third group: x7, x8, x9) with covariance matrices

$$\begin{array}{c} \Sigma_{1}=\left[\begin{array}{ccc} 3.40 & -2.75 & -2.00\\ -2.75 & 5.50 & 1.50\\ -2.00 & 1.50 & 1.25 \end{array}\right],\;\Sigma_{2}=\left[\begin{array}{ccc} 1.0 & 0.5 & 0.3\\ 0.5 & 0.5 & 0.3\\ 0.3 & 0.3 & 0.3 \end{array}\right],\;\Sigma_{3}=\left[\begin{array}{ccc} 1.40 & -2.75 & -2.00\\ -2.75 & 5.50 & -1.00\\ -2.00 & -1.00 & 3.25 \end{array}\right]. \end{array}$$

In this figure, a circular pattern indicates the data sets are independent (or weakly correlated), whereas an elongated pattern shows strong correlation among them, either positive or negative depending on the orientation of the pattern. For example, in Fig 5(a), data sets x8 and x9 are weakly correlated and thus one would not expect to see a connection between them in the inferred network. In contrast, since data sets x1 and x3 are strongly anti-correlated, one would expect to see a connection in the inferred network.

This is a case where there is a distinction between correlated and non-correlated pairs as the data have been constructed as such. This can then be exploited to set the threshold *τ* to identify connectivity in terms of the correlated pairs. Particularly, we set *τ* for ${\bar{\text{MIR}}}_{XY}$ so as to depict all correlated pairs of nodes in the data. The results in Fig 5(c) show that ${\bar{\text{MIR}}}_{XY}$ can be used to successfully infer the network from correlated normal variates. One can see that the data are successfully classified into three distinct groups, and that, the connection between nodes x8 and x9 is missing as they are weakly correlated and the connection between nodes x1 and x3 is present as they are strongly anti-correlated. Since this pair is also the strongest correlated of all, its ${\bar{\text{MIR}}}_{XY}$ value is also maximal and corresponds to the highest bar in Fig 5(b), which is equal to 1.

Here, we have shown that the ${\bar{\text{MIR}}}_{XY}$ can depict the correct number and pairs of correlated data and thus infer successfully the underlying network structure. Since the global financial-markets data of different currency areas are also found to be correlated [43], we will use a similar approach in the next section to infer the network structure of currency exchange rates to the US dollar (USD) and stock indices for 15 currency areas.

## Application to financial-markets data

So far, we have demonstrated the applicability of the method to infer successfully the network structure for artificial data, and we now use it to infer the connectivity in networks of financial-markets data.

Particularly, we have applied the proposed method to infer the financial relations among 15 currency areas using the currency exchange rates to USD and stock indices. The information for the local currencies and stock indices for the 15 currency areas is shown in Table 1. The data used are daily exchange rates of the local currencies to USD, from January 2000 to August 2016 (taken from Datastream, Thomson Reuters database) and stock-index data, from January 2000 to December 2016 (taken from Bloomberg). We have transformed the daily data points, *p*_*t*_ from exchange rates and stock indices to log-return values, *r*_*t*_ = ln(*p*_*t*_/*p*_*t*−1_), where *t* is the index of the data point in the time-series, as this is a common practice in Quantitative Finance [44].

**Table 1:** The local currencies and stock-indices for the 15 currency areas. The first column is the node labels, the second the 15 currency areas, the third the names of the local currency exchange rates with USD and the fourth the stock indices. Notice that the first column is the node label seen in the networks in Fig 6(e) and 6(f).

Node Label	Currency Area	Currency Exchange Rate with USD	Stock Index
1	JPN—Japan	Yen (JPY)	Nikkei 225
2	EU—European Union	Euro (EUR)	Euro Stoxx 50
3	CAN—Canada	Canadian Dollar (CAD)	S&P/TSX Composite Index
4	TWN—Taiwan	New Taiwan Dollar (TWD)	TSEC weighted Index
5	CHE—Switzerland	Swiss Franc (CHF)	SMI Index
6	IND—India	Indian Rupee (INR)	Bombay BSE 30
7	KOR—South Korea	Won (KRW)	Seoul Composite KS11
8	BRA—Brazil	Brazilian Real (BRL)	Bovespa
9	MEX—Mexico	Mexican Peso (MXN)	MXX Bolsa Index
10	NOR—Norway	Norwegian Krone (NOK)	Oslo OBX Index
11	SWE—Sweden	Swedish Krona (SEK)	OMX Stockholm 30 Index
12	SGP—Singapore	Singapore Dollar (SGP)	Straits Times Index
13	ZAF—South Africa	Rand (ZAR)	FTSE/JSE All-Share Index
14	THA—Thailand	Thai Baht (THB)	SET Index
15	DNK—Denmark	Danish Krone (DKK)	OMX Copenhagen 20

**Fig 6:**
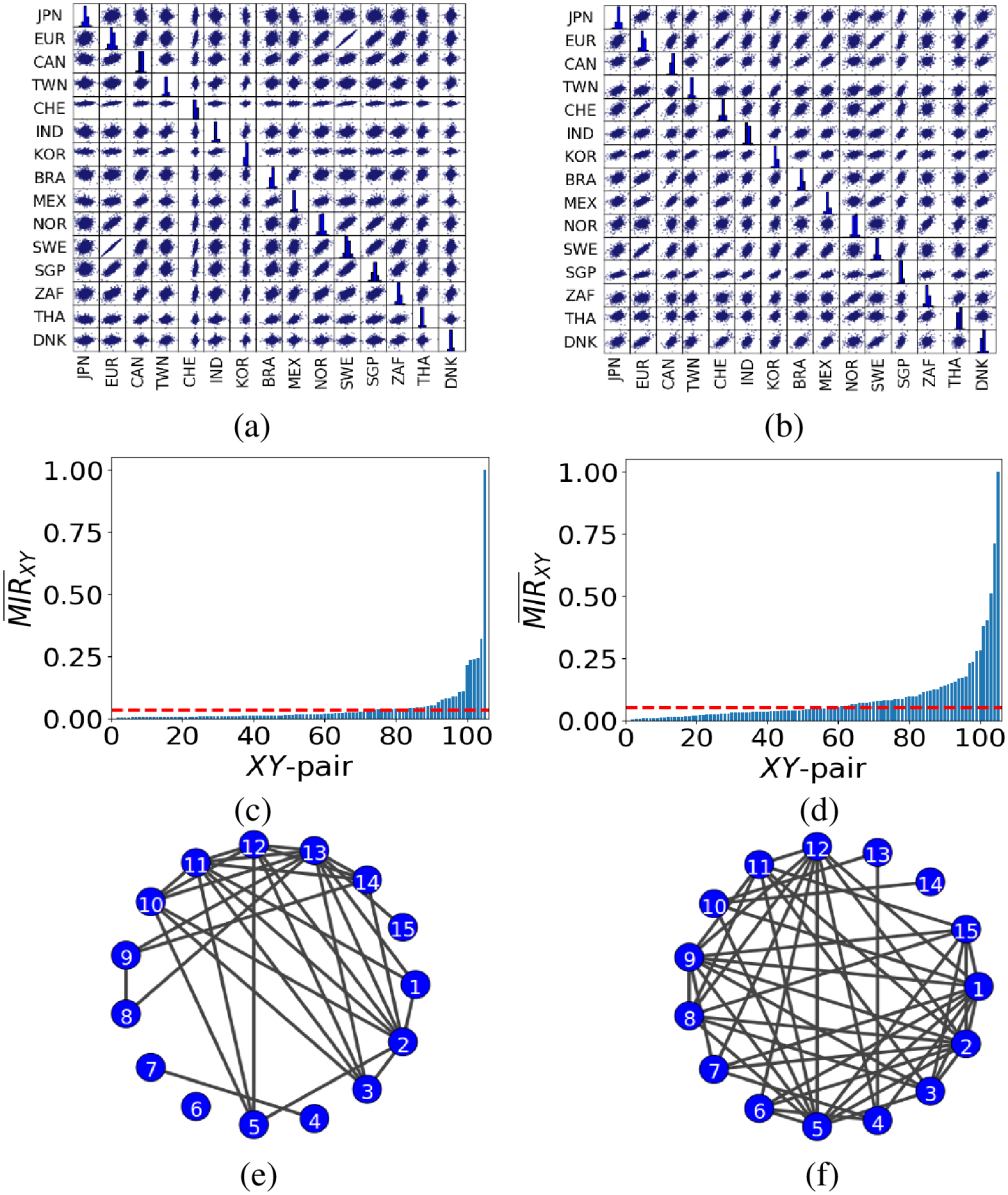
Scatter plots and inferred networks for the financial-markets data. Panels (a), (c) and (e) show the scatter matrix, ordered ${\bar{\text{MIR}}}_{XY}$ values and network of the 15 currency exchange rates. Panels (b), (d) and (f) similarly for the 15 stock indices. The red dash line in (c) corresponds to *τ* ≈ 0.03 and in (d) to *τ* ≈ 0.05. Note that in panels (e) and (f), the nodes represent the currency exchange rates and stock indices for the 15 currency areas, respectively, as in Table 1.

Fig 6(a) and 6(b) present the scatter plots for the daily log-returns of the currency exchange rates and stock-indices, respectively, based on the 15 currency areas. In both plots, the strongest correlated pair is the EU-Sweden (EU-SWE) pair. This strong interrelation is manifested by the highest bars in the ordered ${\bar{\text{MIR}}}_{XY}$ plots in Fig 6(c) and 6(d). The second highest, and rest of the bars in Fig 6(c) do not correspond to the same pairs in Fig 6(d), indicating that the connectivity in the two inferred networks for the currency exchange rates and stock indices is different.

To infer the networks from the ${\bar{\text{MIR}}}_{XY}$ plots, we need to set appropriately *τ* in Fig 6(c) and 6(d). Since, for the financial-markets data there is no clear-cut distinction between correlated and non-correlated pairs (see Fig 6(a) and 6(b)), we use the same idea as for the indirect information exchange data and introduce an additional pair of unidirectional interacting nodes with chaotic logistic map dynamics (see Eq (18) where *r* = 4). The pair is the same for both the currency exchange rates and stock-indices data and is disconnected from the networks to avoid spurious interactions. Again, we assume that any pair of nodes with ${\bar{\text{MIR}}}_{XY}$ bigger than the ${\bar{\text{MIR}}}_{XY}$ of the unidirectional interacting nodes can be regarded as connected. Following this approach, we have found that *τ* ≈ 0.03 and 0.05 for the currency exchange rates and stock-indices data, respectively. The difference in the two thresholds comes from the normalisation in Eq (16) as the maximum values of ${\bar{\text{MIR}}}_{XY}$ for the currency exchange rates and stock-indices are different and they represent information exchange in different networks. Subsequently, in Fig 6(e) and 6(f), we present the resulting inferred networks for the currency exchange rates and stock-indices based on the ${\bar{\text{MIR}}}_{XY}$ values for the 105 (i.e. 15(15 − 1)/2) unique pairs. Doing so, we have been able to infer most of the weakly and all of the strongly correlated pairs of currency areas in both data sets.

Further on, we have performed an analysis to shed light on the structural properties of the two inferred networks. Particularly, we have found that both are small-world networks with small-world measures [19, 45] *σ* ≈ 5 for the exchange rates and *σ* ≈ 3 for the stock-indices, respectively. The higher is *σ* from unity, the better it displays the small-world property, with values of *σ* < 1 indicating a random network. In our case, we found that the stock-indices inferred network is closer to a random network than the currency exchange rates inferred network. We have also found that the latter is dissasortative (mixing by degree) [26] with the coefficient of assortativity *r* ≈ −0.17, whereas the stock-indices inferred network is assortative with *r* ≈ 0.1. Assortative mixing by degree is the tendency of nodes with high degree to connect to others with high degree, and similarly for low degree, whereas dissasortative mixing by degree is the tendency of nodes with high degree to connect to nodes with low degree [26]. This is a qualitative difference between the two inferred networks as economies well-connected in terms of their currency exchange rates prefer to connect with economies less well-connected in terms of the exchange rates as opposed to the behaviour of stock-indices, where the preference is toward the well-connected nodes! Interestingly, our study reveals that there are 32 bidirectional connections with non-trivial information exchange in the currency exchange rates network and 49 in the stock-indices network. Moreover, both networks have a relatively small modularity [46] (i.e. *Q* ≈ 0.12 and *Q* ≈ 0.14 for the currency exchange rates and stock indices, respectively) indicating that the strength of division of both networks into modules (or groups of well-connected economies) is small. Both networks have sparse connections between the nodes within modules and denser connections between nodes in different modules, which shows that global economies tend to connect with other economies world-wide, rather than creating small groups of local economies.

The results in Fig 6(e) show that the Indian Rupee is not connected to any currency because it is not a fully convertible currency, cannot be freely traded on a forex market and requires regulatory approvals for higher-amount transactions. Similar results are evident in other non-convertible (or blocked) currencies, namely the South Korean Won, Taiwan Dollar and Brazilian Real. This is because these currencies are not openly traded on a forex market, generally as a result of government restrictions. Interestingly, our results reveal that the South Korean Won has no connection with other currencies, except with Taiwan Dollar, a neighbour currency. Emerging market currencies, especially those in the same regions, tend to mirror each other [47]. The Brazilian Real and the Mexican Peso have come to epitomise this kind of relationship. Other than that, the Brazilian Real is also connected to the South Africa Rand, a fully convertible currency with excellent connection with most currencies around the world.

According to our results, the Euro is the predominant currency in Europe, including countries not in the European Union, such as Norway and Switzerland. Two of the three Nordic currencies in our study, namely the Swedish and Norwegian Krone, together with the Swiss Franc, form a network of connections among others and the Euro. The Euro-Swedish Krone pair has the highest ${\bar{\text{MIR}}}_{XY}$ value, close to 1. All Nordic countries considered in this study, along with Switzerland, are nonetheless intrinsically linked to the European socio-economic and political situations.

Our results confirm the findings of past studies [48, 49], that most of the world’s major stock indices are integrated into international markets. Fig 6(f) shows that some of the largest stock-indices (by market capitalisation), i.e. Nikkei 225 (Japan), Euro Stoxx 50 (European Union) and SMI Index (Switzerland) are connected with most of the other stock indices. The Euro Stoxx 50 and OMX Stockholm 30 Index (Sweden) pair of stock indices has the closest ${\bar{\text{MIR}}}_{XY}$ value to 1, and hence, share the biggest rate of information exchange. The Straits Time Index (Singapore) is also well connected internationally with other stock indices, because it is one of the world’s most diversified benchmark indices with a mix of stocks that are both domestic and globally focused. The results also show that the SET Index (Thailand) is only connected with one stock index. This is not surprising as the authors in [50] found no co-integration between the stock indices of Thailand and its major trading partners.

The inferred networks of currency exchange rates and stock-indices are relevant to risk managers to use as an investment strategy in portfolio management, as they give an indication of the mix of assets to hold in order to form a well-diversified portfolio. However, there are limitations to this, e.g. during periods of financial crisis, as diversification does not hold in times of financial stress [51, 52].

## Conclusions

In this paper, we used the normalised Mutual Information Rate to infer the network structure in artificial and financial-markets data of 15 currency areas including the EU, from 2000 to 2016. Specifically, we showed how the underlying network connectivity among the nodes of financial time-series data, such as foreign currency exchange-rates and stock-indices can be inferred. We first demonstrated the applicability of the method by applying it to artificial data from chaotic dynamics and to cases of correlated normal variates. Our results for the artificial data showed that the method can be used to successfully infer the underlying network structures. This uses the data recorded from the coupled dynamics and assumes no previous knowledge of the adjacency matrices, other than to estimate the percentage of successful network inference.

We then applied the method to infer the underlying connectivity of currency exchange rates and stock-index data from the 15 currency areas, and performed an analysis of both inferred networks to identify their structural properties. We found that both are small-world networks, with the stock-indices network being assortative by mixing degree and closer to a random network than the currency exchange-rates network, which was found to be dissasortative by mixing degree. This is a qualitative difference between the two inferred networks and shows economies which are well-connected in terms of their currency exchange rates prefer to connect with economies less well-connected in these terms. This contrasts with the behaviour of stock-indices, where the preference is toward the well-connected nodes! Interestingly, our study revealed that both inferred networks have relatively small modularities, which shows that global economies tend to connect with other economies world-wide rather than creating smaller groups of well-connected local economies.

Finally, our analysis showed that the normalised Mutual Information Rate is a mathematical method that can be used to infer the network structure in complex systems. It is a method of estimating the amount of information exchanged among nodes in systems such as financial markets and identifying their connectivity. In our study, the method allowed us to infer two networks and show how the currency areas are connected to each other. The currency exchange-rates and stock-indices networks are relevant for risk managers to use as an investment strategy in portfolio management as they give an indication of the mix of assets to hold in order to form a well-diversified portfolio.
